# Spinal dysraphism in adulthood: do they have different symptoms and outcomes? Evaluating the impact on the Quality of Life and the need for transitional care from pediatric to adult population

**DOI:** 10.1007/s00381-025-07073-z

**Published:** 2025-12-03

**Authors:** Davide Palombi, Mariapia Masino, Gabriele Ciaffi, Timothee de Saint Denis, Claudia Rendeli, Gianpiero Tamburrini, Luca Massimi

**Affiliations:** 1 Department of Pediatric Neurosurgery, Fondazione Policlinico Universitario Agostino Gemelli IRCCS, Rome, Italy; 2 Department of Neuroscience, Università Cattolica del Sacro Cuore, 00168 Rome, Italy; 3 Department of Pediatrics, the University of Campania ″L. Vanvitelli″, Naples, Italy; 4 Department of Pediatric Orthopedic and Reconstructive Surgery, Armand Trousseau Hospital, AP-HP, Sorbonne University, Paris, France; 5 Spina Bifida and Malformative Uropathies Centre, Department of Women’s and Children’, Health Sciences and Public Health, ″Agostino Gemelli″ University Polyclinic Foundation - IRCCS, L.Go A. Gemelli 8, 00168 Rome, Italy

**Keywords:** Spina bifida, Spinal lipomas, Dysraphism

## Abstract

**Introduction:**

Spinal lipomas are common forms of closed spinal dysraphism, typically diagnosed in childhood but sometimes remaining unrecognized until adulthood. Adult presentation often includes pain, sensory disturbances, and urogenital dysfunction. While pediatric surgical management is well studied, adult outcomes, especially psychosocial ones, remain poorly understood. This study evaluates surgical, neurological, and psychosocial outcomes in adults undergoing their first surgery, comparing them with those of patients treated in childhood and followed into adulthood. It also emphasizes the importance of transitional care across the lifespan.

**Methods:**

This retrospective cohort study included 24 patients treated at a single institution: 12 adults (SDA) undergoing first-time surgery from 2022 to 2023, and 12 historical controls (PSD) treated in childhood (2000 to 2005). Pre- and postoperative data were collected using a 14-item questionnaire assessing neurological, functional, and psychosocial outcomes. Statistical comparisons were performed within and between groups.

**Results:**

The median age at surgery was 30 years in SDA vs. 2 years in PSD. 75% of SDA patients had never been previously diagnosed with spinal dysraphism. Only urinary continence showed significant improvement neurologically in SDA patients (*p* = 0.025). However, psychosocial outcomes including pain, quality of life, social participation, and self-esteem, significantly improved postoperatively in the adult group. No surgical complications occurred in SDA; two CSF leaks were observed in PSD. Comparison of long-term outcomes revealed similar results across groups, except for a higher level of self-esteem in PSD patients (*p* = 0.039).

**Conclusion:**

Surgical treatment of spinal lipomas in adulthood can provide meaningful symptomatic and psychosocial benefits, even without gross-total resection. This study underscores the limitations of viewing spinal dysraphism through a strict pediatric-adult binary and highlights the need for individualized, stage-specific care. The findings advocate for structured, lifelong transitional care pathways that integrate psychological and social support, addressing the full spectrum of patient well-being.

## Introduction

Spinal lipomas are among the most common forms of closed spinal dysraphism, typically diagnosed in the pediatric population, with an estimated incidence of 0.3–0.6 per 10,000 live births [1–3]. These lesions are frequently associated with tethered cord syndrome (TCS) and may lead to progressive neurological decline if not surgically addressed [4–7].

Although spinal lipomas are congenital, their clinical manifestation may be delayed until adulthood, often triggered by trauma, physical exertion, pregnancy, or unidentified causes [8–10]. Adults typically present with low back and radicular pain, sensory deficits, and urinary or bowel dysfunction; motor weakness and sexual disturbances are less frequent [11–13]. These symptoms do not often emerge during childhood, resulting in a delayed diagnosis of congenital spinal dysraphism [4, 14, 15].

Presentation, classification, and surgical management in children have been extensively studied, with several large series contributing to the current practice patterns and consensus on surgical indications [3, 16–19].

Despite increasing reports on spinal dysraphism and adult tethered cord syndrome (ATCS), there is no consensus regarding surgical indications, timing, or outcomes in adulthood. Several studies have documented clear postoperative improvements, particularly in pain relief and neurological stability [7, 13, 18, 20]. However, others highlight a limited benefit or higher complication rates, especially in cases with prolonged symptom duration or those requiring revision surgery [2, 5, 21, 22].

Although children with spinal dysraphism are typically managed within structured, multidisciplinary teams, this continuity of care frequently disappears upon transition to adulthood [15, 23–25]. The absence of coordinated follow-up can lead to delayed recognition of clinical deterioration, missed opportunities for early intervention, and ultimately, loss to follow-up [15, 25]. These gaps highlight the need for transitional pathways between pediatric and adult neurosurgical services [24].

Notably, while previous studies have focused primarily on neurological and functional outcomes, the emotional and psychological aspects of spinal dysraphism have remained largely unexplored [22]. To date, no studies have systematically evaluated the impact of this condition and its treatment on self-esteem, social interaction, and professional life, crucial aspects of the daily lives of individuals affected by it.

Therefore, this study aims to highlight the importance of assessing not only surgical and neurological outcomes but also the psychological impact of the disease by evaluating pre- and post-operatively adults undergoing first-time surgery and comparing them to patients who were operated on in childhood and are now followed into adulthood.

## Materials and methods

### Study design and setting

This retrospective, observational cohort study was conducted at the Pediatric Neurosurgery Department of Policlinico Universitario A. Gemelli IRCCS, Rome, Italy. The study includes patients who underwent surgery between January 2000 and December 2023 and adheres to the STROBE guidelines for observational research [26].

### Participants

Two groups of patients with spinal dysraphism were included: 1) consecutive cases who underwent their first-time surgical treatment for spinal lipomas with tethered cord in adulthood (SDA) from 2022 to 2023. Cases were collected from 2022 since, from that time, a Transitional Care Program for Spina Bifida was started in our Institution and pediatric neurosurgeons were allowed to take care also of new adult patients with dysraphism; and 2) consecutive controls represented by adult patients operated on during childhood (PSD) from 2000 to 2005. This period was selected to collect consecutive controls with matching genre, spinal dysraphism, surgical strategy, and methods, ensuring a sufficiently long follow-up to assess long-term outcomes.

### Eligibility criteria

The inclusion criteria were radiologically and clinically confirmed closed spinal dysraphism, surgical treatment at our center, a minimum follow-up of 12 months, availability of information on clinical and neurological outcomes, and quality of life. Exclusion criteria were open spinal dysraphism, isolated filum terminale sectioning, previous or multiple surgeries, incomplete or missing preoperative and postoperative data.

### Data collection and variables

Data was collected retrospectively from institutional electronic medical records and via a custom 14-item questionnaire. The following variables were analyzed: age at surgery, sex, and length of follow-up. Neurological, pain-related, and quality of life (QoL) outcomes were assessed both pre- and postoperatively. Scales ranged from 0 to 10, with higher values reflecting more severe symptoms or greater functional limitation and dysraphism’s impact in the domain, except for QoL and social life (where higher values indicated better outcomes). Urinary control was assessed in all patients using the assigned questionnaire, and in the PSD group, a dedicated pediatric urological evaluation, including bladder ultrasound and urodynamic testing, was performed.

All questionnaire data were collected during outpatient visits or phone interviews conducted by the treating team. To ensure comparability, the same evaluation protocol was applied to both groups.

### Outcome measures

The primary outcome was assessed by comparing patient-reported QoL, pain, and social/work functioning before and after surgery in the SDA group. The secondary outcome was evaluated by comparing the postoperative neurological and psychosocial status between the SDA and PSD groups at the last follow-up.

### Statistical analysis

Descriptive statistics were calculated for all variables. Continuous variables are reported as mean ± standard deviation. Paired Student’s t-test or Wilcoxon signed-rank test was used to compare pre- and post-operative values within the SDA group. The Mann–Whitney U test was used to compare continuous outcomes between the SDA and PSD groups. The chi-square test was used for categorical comparisons, including the presence or absence of postoperative complications. A *p*-value < 0.05 was considered statistically significant. All analyses were performed using SPSS (IBM SPSS Statistics for Windows, Version 25.0).

### Study size

The sample size reflects all eligible cases surgically treated at our institution since the Spina Bifida Transitional Care program was instituted. The PSD group is constituted by consecutive patients with an actual average age comparable to the SDA group.

### Surgical setting

The same neurosurgical team treated all patients under standardized operative protocols, including intraoperative neurophysiological monitoring (IONM) [20, 27]. Surgical indications were based on progressive neurological symptoms, pain, or dysfunction attributable to tethered cord syndrome and/or spinal dysraphism. Out of 12 patients, progressive pain (8/12, 75%) and lower limb deficit worsening (4/12, 25%) were the neurological deficits for whom surgery was needed.

All procedures were performed under general anesthesia with the patient in the prone position and continuous intraoperative neurophysiological monitoring. A standard posterior midline approach was employed. In most cases, a laminectomy or laminotomy was performed at one or more levels rostral to the dysraphic segment to achieve adequate exposure. Dissection proceeded under microscopic magnification, with meticulous adhesiolysis to isolate and subtotal debulk the lipomatous tissue compressing the neural placode. When present, a thickened filum terminale was transected to complete the detethering. Dural closure was reinforced with synthetic grafts and fibrin glue, and multilayered wound closure was performed. In cases with significant soft tissue involvement, plastic surgeons were required, and techniques such as lipectomy and adipofascial flap advancement were used to ensure adequate dural coverage and skin closure. No patients in this series exhibited clinical or radiological signs of retethering at follow-up.

## Results

### Study population

The study cohort included 24 patients divided equally into two groups: 12 individuals who underwent surgery in adulthood (SDA group) and 12 patients who were operated on in childhood and followed into adulthood (PSD group). The median age at surgery was significantly higher in the SDA group (30 years; SD ± 9.47) compared to the PSD group (2 years; SD ± 3.44; *p* < 0.0001), although the actual age of the PSD group (24.93 years, SD ± 3.95) was similar to that of the SDA group. Sex distribution was comparable across groups, with a slight predominance of females in both (SDA: 7 females, 4 males; PSD: 8 females, 4 males; *p* < 0.0001). Regarding spinal dysraphism subtypes, the SDA group included 6 cases of lipomyelomeningocele (LMC), while the PSD group consisted of 5 LMCs. The median follow-up period was markedly shorter in the SDA group (2 years; SD ± 0.83) than in the PSD group (22.5 years; SD ± 3.29; *p* < 0.0001). These demographic differences highlight the distinct clinical trajectories of patients treated in childhood versus those undergoing intervention in adulthood.

Table 1 summarizes patient demographic data, the type of dysraphism, the age at surgery, and the duration of follow-up.

**Table 1:** Patients’ demographics

Parameters	SDA (N = 12)	PSD (*N* = 12)	*p*-value
Age (years, mean, SD, range)	30 (± 9.47)	2 (± 3.44)	< 0. 0001
Male/Female	F = 7; M = 4	F = 8; M = 4	
Spinal Dysraphism	LMC 6 Lipomas 6	LMC 5 Lipomas 7	
Follow-up (months)	2 (± 0.83)	22.5 (± 3.29)	< 0.0001

### Neurological function in the SDA group

Table 2 reports pre- and post-operative neurological outcomes in the SDA group. A statistically significant improvement was observed only in urinary continence (*p* = 0.025). Strength, sensibility, fecal continence, and sexual function tended to improve but did not reach significance.

**Table 2:** Neurological Pre-operative and post-operative values for the SDA (Spinal Dysraphism operated in Adulthood) group

Pre-operative						Post-Operative				
ID	Strength & Movement	Sensibility	Urinary Control	Fecal Continence	Sexual function	Strength & Movement	Sensibility	Urinary Control	Fecal Continence	Sexual Function
1	6	5	0	8	10	6	5	0	8	10
2	4	2	2	4	4	8	8	9	8	8
3	6	3	0	10	10	10	8	10	10	10
4	3	0	5	5	9	6	3	5	10	10
5	5	6	10	9	8	6	7	10	9	8
6	5	8	4	5	7	8	9	3	3	6
7	10	10	2	10	10	10	10	8	10	10
8	10	8	7	4	0	10	8	7	4	0
9	10	10	8	9	8	10	10	8	9	8
10	7	10	1	5	6	7	10	3	8	6
11	6	9	5	10	8	3	3	8	10	6
12	9	7	0	7	1	9	7	5	7	4
Total	6.75 ± 2.45	6.5 ± 3.37	3.67 ± 3.93	7.17 ± 2.44	6.75 ± 3.41	7.75 ± 2.22	7.33 ± 2.49	6.33 ± 3.14	8 ± 2.33	7.17 ± 3.01
*p*-value						0.125	0.358	**0.025**	0.184	0.392

### Quality of life and pain management: pre- vs post-operative (SDA)

**Fig. 1:**
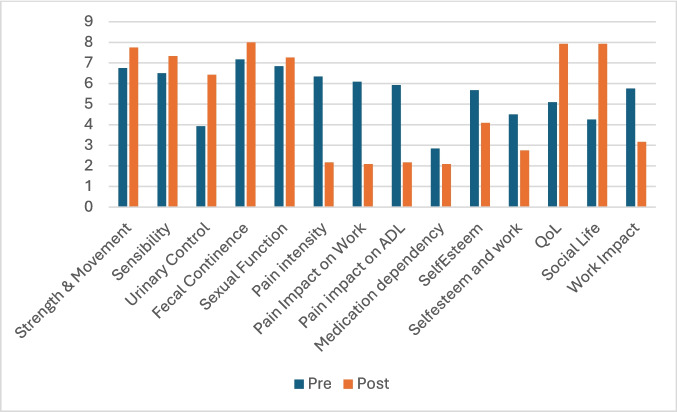
Clustered bar chart illustrating the pre- and post-operative mean scores across a range of clinical and psychosocial domains in the SDA group. Blue bars represent preoperative values, while orange bars represent postoperative values. In neurological function variables, urinary function was the only variable that improved significantly, while in the psychological and pain management group, only medication dependency didn’t improve significantly (*p* = 0.445)

As shown in Table 3 and Fig. 1, patients in the SDA group reported significant improvements across nearly all psychosocial and pain-related domains after surgery. The most notable changes occurred in pain intensity, its impact on work and daily life, quality of life, social participation, and self-esteem. Medication dependency was the only domain that did not show significant change (*p* = 0.445).

**Table 3:** Quality of life and pain management and influence pre-operative and post-operative values for the SDA (Spinal Dysraphism operated in Adulthood) group

ID	Pre-operative									Post-Operative								
	Pain intensit y	Pain Impact on Work	Painimpact on ADL	Medica tion depend ency	SelfEst eem	Self-esteem and work	QoL	Social Life	Work Impact	Pain intensit y	Pain Impact on Work	Pain impact on ADL	Medica tion depend ency	SelfEst eem	Self-esteem and work	QoL	Social Life	Work Impact
1	8	8	8	6	8	7	4	5	8	2	0	0	0	8	8	9	8	3
2	8	8	8	5	5	5	5	4	5	2	4	4	1	5	5	8	8	4
3	9	7	5	0	5	7	5	0	5	0	0	0	0	0	0	10	10	10
4	8	10	10	0	4	4	5	5	5	0	0	0	0	0	0	8	8	2
5	8	10	10	5	6	0	6	7	6	8	6	7	2	0	0	6	7	7
6	9	9	9	0	9	9	2	5	7	4	4	5	10	8	3	8	9	2
7	9	8	8	4	0	0	5	7	9	0	0	0	0	0	0	8	5	2
8	6	3	3	0	10	0	7	2	0	2	0	0	0	8	0	8	10	0
9	0	0	0	0	0	0	7	2	0	0	0	0	0	0	0	7	5	0
10	7	7	7	7	4	4	5	5	6	3	3	3	4	3	3	7	8	2
11	0	0	0	0	9	5	8	7	6	0	0	0	0	8	5	8	8	2
12	2	1	1	1	6	9	2	0	10	0	0	0	0	4	3	8	9	2
Total	6.17±3.46	6.17±3.46	5.75±3.81	2.33±2.8	5.5±3.26	4.17±3.48	5.08±1.83	4.08±2.53	5.58±3.05	1.75±2.41	1.42±2.19	1.58±2.5	1.42±2.96	3.67±3.62	2.25±2.7	7.92±0.99	7.92±1.62	3±2.86
*p*-value	**0.001**	**0.001**	**0.001**	0.445	**0.011**	**0.045**	**0.001**	**0.004**	**0.004**	**0.001**	**0.004**	**0.004**	**0.001**	**0.004**	**0.004**	**0.004**	**0.004**	**0.004**

### Complications

No complications occurred in this cohort, except for 2 CSF leakages in the PSD group (16.6%), as shown in Table 4.

**Table 4:** Complications

Complications	SDA	PSD
Total Surgical Site Infections	0 (0%)	2 (16.66%) (%)
CSF leak	0 (0%)	2 (100%)

### SDA vs PSD group: long-term outcomes

**Fig. 2:**
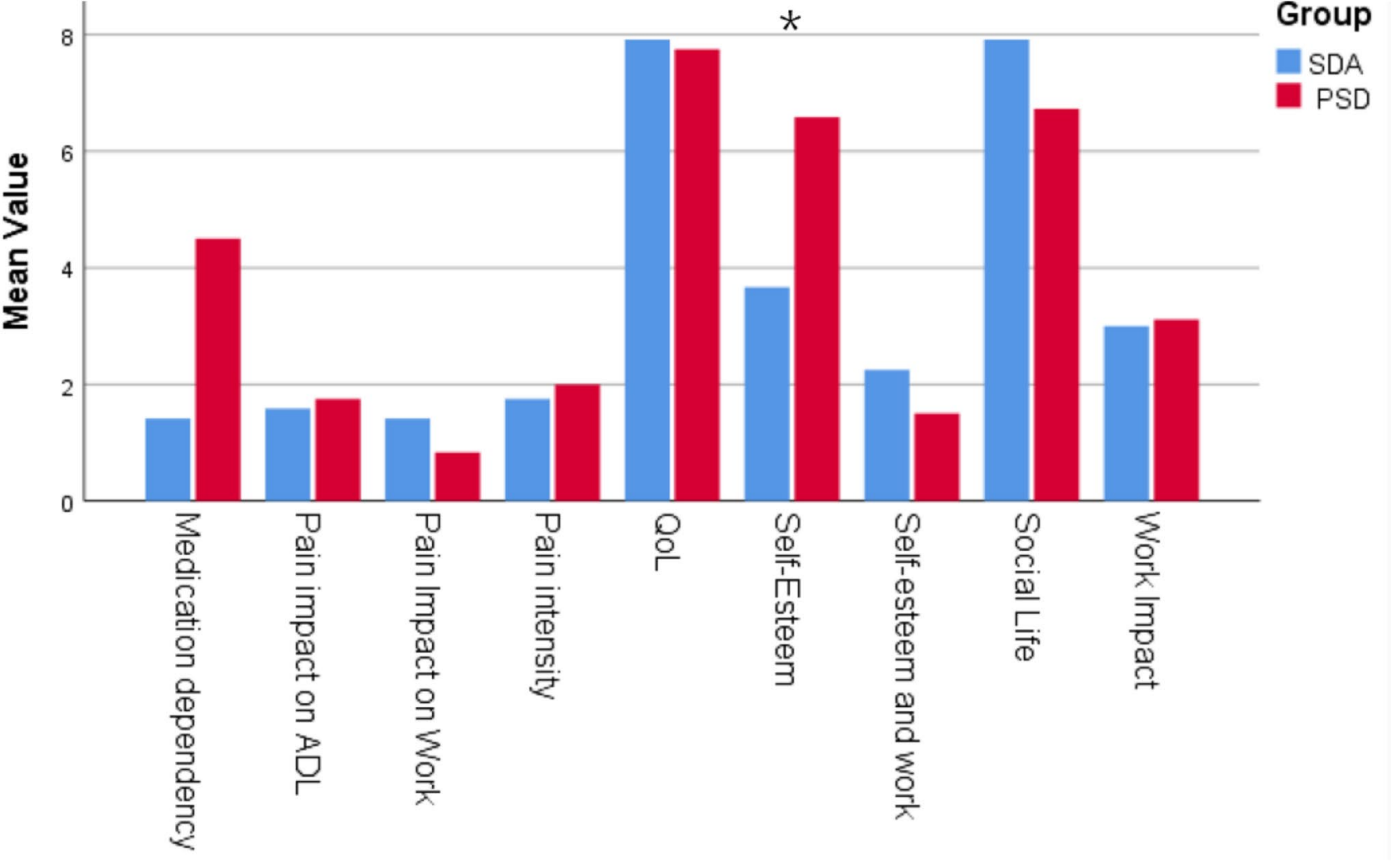
A clustered bar chart illustrates the post-operative mean scores of psychosocial domains in the SDA vs PSD groups. *Blue* bars represent SDA postoperative values, while red bars represent PSD ones. Only self-esteem showed a difference between the 2 groups (*p* = 0.039)

Postoperative scores between the SDA and PSD groups were compared and are detailed in Table 5 and Fig. 2. Overall, outcomes were comparable in both groups. The only statistically significant difference was found in self-esteem, which was higher in the PSD group (*p* = 0.039).

### Urinary and bowel function (SDA and PSD)

Concerning the sphincteric function, in the SDA group, urinary control was severely impaired preoperatively, with several patients requiring intermittent catheterization and one patient with a permanent catheter. After surgery, urinary function significantly improved in most cases, including the conversion from a permanent catheter to intermittent catheterization (*p* = 0.025). Fecal continence also showed a trend toward improvement, although it did not reach statistical significance (*p* = 0.184). In the PSD group, almost all patients had preserved urinary and fecal continence at the time of adult evaluation, except for one case requiring intermittent catheterization since childhood and another under medical therapy (oxybutynin). Importantly, no patients in the PSD group experienced progressive deterioration with growth Table 5.

**Table 5:** Neurological function, quality of life, pain management, and influence post-operative values for the PSD (Pediatric Spinal Dysraphism) group

ID	Neurological Function					Quality of Life and Pain Management								
	Strength & Movement	Sensibility	Urinary continence	Fecal continence	Sexual Function	Pain intensity	Pain Impact on Work	Pain impact on ADL	Medication dependency	Self-Esteem	Self-esteem and work	QoL	Social Life	Work Impact
1	10	10	10	10	10	0	0	0	0	5	0	10	5	2
2	10	10	10	10	10	0	0	0	0	0	0	8	5	2
3	8	10	10	10	0	6	0	9	0	10	4	10	5	NA
4	8	5	10	10	0	10	10	9	10	10	5	8	3	2
5	6	6	6	1	7	0	0	0	10	8	2	7	NA	4
6	10	9	8	9	NA	0	0	0	4	5	0	7	7	NA
7	10	10	8	10	0	2	0	3	10	8	0	9	8	3
8	7	8	9	9	10	0	0	0	10	5	0	10	10	3
9	7	2	9	6	9	0	0	0	0	9	2	7	6	3
10	10	10	10	10	10	6	0	0	0	7	0	10	10	NA
11	10	7	0	10	NA	0	0	0	10	7	0	7	5	4
12	10	10	10	10	NA	0	0	0	0	5	5	0	10	5
Total	8.83± 1.53	8.08± 2.61	8.33± 2.9	8.75± 2.7	6.22± 4.76	2± 3.41	0.83± 2.88	1.75± 3.49	4.5± 4.98	6.58± 2.81	1.5± 2.06	7.75± 2.76	6.72± 2.45	3.11± 1.05
*p*-value	0.18	0.48	0.12	0.47	0.61	0.83	0.58	0.89	0.08	**0.039**	0.45	0.84	0.19	**0.903**

## Discussion

Despite decades of surgical experience with spinal lipomas in children, their evolution across the lifespan, and particularly into adulthood, remains poorly understood. While much of the literature focuses on indications in children or early intervention and anatomical correction, relatively little is known about how these patients live as adults [16–18]: how they live with residual symptoms, how the condition affects their quality of life, and what role late surgical intervention can still play [22, 28].

This study offers a unique perspective on this trajectory by comparing adults who underwent spinal lipoma surgery in adulthood with those treated in childhood and followed into maturity. What emerges is that the patient experience in adulthood diverges significantly from the pediatric. Surgical timing, indications, expectations, and outcomes are derived not only from anatomy and imaging but also from the symptoms that accumulate and evolve.

### Which symptom is the symptom?

The decision to operate in adulthood is rarely an incidental one. Unlike in pediatrics, where prophylactic surgery or surgery driven by objective imaging findings is proposed, adults may arrive at surgery after months or years of progressive, life-disrupting symptoms [2, 16, 19, 29]. These usually include low back or radicular pain, sensory changes, and urogenital dysfunction, as described in the literature [8–10]. While severe motor deficits and gait disturbances are less common, subtle functional limitations are frequent and can significantly impact a patient’s independence and quality of life [11, 13].

**Fig. 3:**
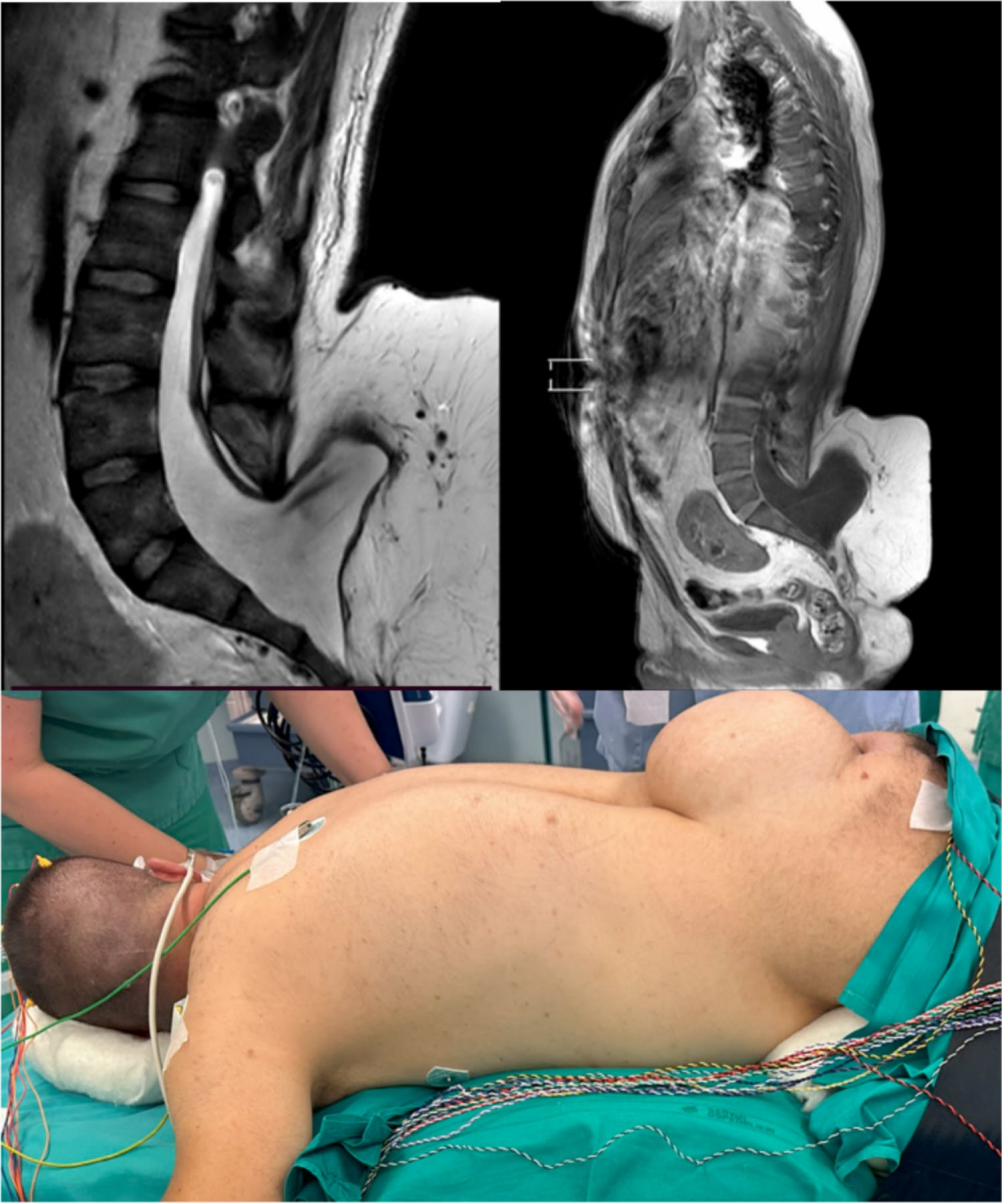
Illustrative case of the SDA group. A 40-year-old male with a history of untreated congenital lipomyelomeningocele presented with progressive gait disturbance and longstanding urinary incontinence requiring intermittent catheterization. Neurological examination revealed bilateral foot drop, lower limb hypotrophy, and reduced perineal sensation. MRI confirmed spinal dysraphism with tethered cord and a large extrarachidian lipoma. He underwent untethering surgery and partial excision of the lipoma, with IONM. The postoperative course was uneventful, with the resolution of pain related to mass effect without new neurological deficits

In this cohort, the primary driver was pain, which was sometimes poorly localized, sometimes triggered by posture or movement, but almost always affected occupational, social, or emotional functioning [22]. One patient, for example, suffered severe pain each time they lay supine due to a large lipoma’s mass effect (Fig. 3). These stories reflect the complex symptomatology of ATCS, which justifies intervention from the patient’s perspective [7].

### And the outcome?

The surgical philosophy for pediatric spinal lipomas, illustrated in Pang’s work advocating total resection and placode reconstruction to maximize progression-free survival, is not easily translatable to adults [2, 5, 16]. Adults often present with longstanding tethering, dense arachnoid scarring, and mature tissue planes [30]. Attempting radical dissection in this context increases the risk of neurological injury, and it may not be justified, particularly when the primary complaint is pain or urogenital dysfunction rather than progressive motor decline [21].

The surgical strategy for spinal lipomas differs markedly between pediatric and adult populations. In children, Pang’s technique emphasizes the total resection of the lipoma and meticulous reconstruction of the placode to maximize progression-free survival and reduce the risk of retethering. This aggressive approach is facilitated by more pliable tissues, less scarring, and greater anatomical clarity early in life. In contrast, adult patients often present with chronic tethering, dense arachnoid adhesions, and mature, fibrotic tissue planes that significantly increase the risk of neurological injury during radical resection [14, 29]. Consequently, the adult surgical approach described in our series prioritizes a symptom-driven and function-preserving strategy. Subtotal or even partial debulking, targeted untethering, and duraplasty were performed with the aid of intraoperative neurophysiological monitoring to minimize risk while achieving meaningful symptom relief [12, 13, 30]. This paradigm shift highlights the importance of individualizing surgical goals based on patient age, symptomatology, and intraoperative anatomy, rather than prioritizing total resection at all costs.

This study suggests a symptom-driven and function-preserving approach, which may be more suitable for adults. Even partial untethering or decompression, when performed with attention to neurophysiological integrity, has been shown to significantly improve pain intensity, self-esteem, and overall quality of life [13, 18]. In the SDA group, one patient transitioned from a long-term indwelling catheterization to intermittent catheterization postoperatively. In this population, surgical success should not be defined by gross-total resection, but by the relief of disabling symptoms and preservation of function.

Therefore, the key difference between the two populations concerns sphincteric function. In children, surgery primarily aims to prevent deterioration, while in adults it cannot restore long-standing deficits but may stabilize symptoms, relieve pain, and improve psychological outcomes [7, 29]. In our series, SDA patients often had impaired urinary control preoperatively, with partial improvement after surgery, whereas almost all PSD patients maintained good urinary and fecal function into adulthood without progressive deterioration.

### The long-term natural history: do all the asymptomatic lipomas remain asymptomatic?

Another key question is whether these adults could have been operated on earlier, possibly avoiding deterioration. The natural history of asymptomatic spinal lipomas has been a topic of long-standing debate. While some authors suggest a benign course, long-term studies tell a more cautionary tale [1, 31]. Wykes et al. reported deterioration in up to 40% of conservatively managed lipomas over 10 years, especially in transitional types or those associated with syrinxes [1, 31].

In our cohort, 9 out of 12 (75%) adult patients presented with previously unrecognized spinal dysraphism, receiving their first diagnosis only in adulthood following the onset of progressive symptoms such as pain, urinary dysfunction, or neurological decline. The remaining 3 patients had been diagnosed in childhood but were either managed conservatively or lost to structured follow-up. This distribution highlights a long-term management issue with spinal lipomas, namely, the underdiagnosis or inadequate surveillance of these conditions as patients transition from pediatric care [15, 25]. These findings suggest that many adults with newly symptomatic spinal dysraphism are not atypical cases, but instead represent the late, predictable progression of a congenital condition [18]. They underscore the urgent need for a more proactive and continuous monitoring strategy from childhood into adulthood, reinforcing the call for robust transitional care models to prevent avoidable deterioration [32].

### The need for lifelong, structured transitional care

Underlying many of these issues is proof of failure in transitional care. Multidisciplinary pediatric teams typically manage children with spinal dysraphism. However, follow-up often becomes fragmented or absent as they age [15, 23]. Many years ago and more recently, many authors have highlighted the risks of discontinuity in care across several pediatric neurosurgical domains [15, 23–25].

From the multidisciplinary management point of view, the SDA patients, diagnosed only in adulthood, had never been followed by a dedicated bowel or bladder team before surgery, and were usually managed with medical therapy alone. With the introduction of the transitional care program, patients are now systematically followed by a multidisciplinary team that includes urology, rehabilitation, and neurosurgery.

Our findings emphasize that this gap extends not only to the clinical level but also to the psychological level. Beyond continence and mobility, these patients face issues of self-image, emotional well-being, social integration, and professional identity [22]. Standard neurological scales do not capture these elements; however, they are clearly represented in our postoperative questionnaire results. Self-esteem and work-related confidence improved significantly following surgery in this series, often without dramatic neurological changes, except for urinary function, which showed a significant improvement (*p* < 0.05). This suggests that psychosocial outcome measures should be considered core endpoints, not secondary ones, in managing spinal dysraphism.

### Limitations and future directions

This study has some limitations. This is a single-center, retrospective analysis with a relatively small sample size and a non-validated QoL tool, since the Transitional Care program was started only in 2022. Nevertheless, it demonstrates that spinal lipoma surgery can be a safe and beneficial procedure for adult patients, particularly when guided by symptoms and patient-centered goals.

Future research should develop and validate psychosocial outcome instruments specific to spinal dysraphism, exploring long-term functional and emotional trajectories. Transitional care models should be implemented, including psychological and social support components.

## Conclusions

This study highlights the clinical and psychological impact of spinal dysraphism in adulthood, offering new insights into a condition traditionally managed in pediatric settings. Our findings suggest that surgical treatment, tailored to symptom relief rather than gross total resection, can significantly improve pain, quality of life, and self-perception without increasing the risk of complications even in adult patients.

Importantly, this is the first study to systematically explore the emotional and social dimensions of the disease, showing that outcomes such as self-esteem, work ability, and social participation are essential indicators of patient well-being. Often neglected in surgical literature, these aspects should be integrated into future care models. Moreover, our findings emphasize that the traditional binary distinction between pediatric and adult populations may not fully capture the progressive and time-dependent nature of this condition. Instead, a more nuanced, individualized approach, accounting for developmental stages such as adolescence, young adulthood, and middle age, is needed to optimize both clinical and psychosocial outcomes.

Finally, our results reinforce the urgency of implementing structured transitional care pathways that go beyond urological or orthopedic management and include long-term psychological, social, and rehabilitative support tailored to each patient’s evolving needs. Our results underline the urgent need for structured transitional care pathways beyond urological or orthopedic follow-up and incorporating long-term psychological and social support.

## Data Availability

The datasets used and/or analyzed during the current study are not publicly available due on our policy statement of sharing clinical data only on request but are available from the corresponding author on reasonable request.
